# Alcohol Abuse Associated with Accumulated Periods of Precarious Employment: A Four-Year Follow-Up Study of a Young Population in Korea

**DOI:** 10.3390/ijerph19127380

**Published:** 2022-06-16

**Authors:** Sungjin Park, June-Hee Lee, Jongin Lee

**Affiliations:** 1 Department of Occupational and Environmental Medicine, Incheon Nasaret International Hospital, Incheon 21972, Korea; psjin9318@yonsei.ac.kr; 2 Department of Occupational and Environmental Medicine, Soonchunhyang University Seoul Hospital, Seoul 04401, Korea; junelee@sch.ac.kr; 3 Department of Occupational and Environmental Medicine, Seoul St. Mary’s Hospital, College of Medicine, The Catholic University of Korea, 222, Banpo-daero, Seocho-gu, Seoul 06591, Korea

**Keywords:** alcohol abuse, gender, mental health, occupational health, precarious employment

## Abstract

This study aims to explore how precarious employment among young age groups affects alcohol-use disorders. Using samples from Youth Panel 2007, a longitudinal and annual follow-up survey, the association between alcohol-use disorder assessed with CAGE and the accumulated years of precarious employment was assessed with logistic regression analysis. During the 4-year follow-up period, precarious employment for 2–4 years (OR 2.08, 95% CI 1.02–4.24) showed a significantly increased risk of alcohol-use disorder compared with the full-time permanent sustained group. Among young male adults, precarious employment for 2–4 years (OR 2.57, 95% CI 1.07–6.14) also showed a significantly increased risk of alcohol-use disorder, while it was not significant in women (OR 1.51, 95% CI 0.43–5.31). Although the prevalence of alcohol-use disorders was highest in groups with precarious employment for 2–4 years among female young adults, no significant association between alcohol-use disorders and precarious employment was found. This study suggests that the longer the precarious employment, the higher the risk of alcohol-dependence disorder, and showed that the tendency was stronger in males. In addition, because people engaged in precarious employment are vulnerable to alcohol-use disorders, policy programs focusing on them are needed.

## 1. Introduction

Humans spend a considerable proportion of their lives working. It is a well-known fact that psychological factors emanating from work itself can adversely affect mental health. Job stress can be measured by using a variety of tools, including the demand–control model or effort–reward model, of which the validity has been verified in several works of literature [1,2]. Job stress acts as a major risk factor for depression [3], is related to suicidal ideation [4], and causes various mental effects. In addition, it is related to cardiovascular risk factors such as high blood pressure and arteriosclerosis [5]. Moreover, job stress can cause major lifestyle shifts, and lead to social problems such as misuse of drugs and alcoholism; for example, the level of stress accumulated at work can be associated with an increasing amount of alcohol consumed [6]. As such, there is job stress that occurs due to the work itself, and occupational health professionals should be able to mediate the unhealthy effects.

However, for humans who are social beings, work is more than just a livelihood; in general, the status of employment affects mental health. In comparison with other conditions, employed people display lower levels of depression, anxiety, and psychological stress than unemployed or under-employed people [7]. Neurocognitive function in various areas can also be associated with employment status [8]. Therefore, paradoxically, for unstable labor, the possibility that the one’s job will be discontinued can rather worsen the health benefits of employment. Precarious work has continued to expand since the 1970s due to education level (high school or below, which has been a subject of many scientific studies [9]). It is relatively easy to predict that employment conditions will affect mental health. Depression, anxiety, and emotional exhaustion have been reported as adverse effects of unstable labor [10]. Moreover, a recent study has shown that lockdown caused by COVID-19 can adversely affect mental well-being; the impacts on mental health are expected to be greater in precarious jobs, which are easy to be dismissed, as the pandemic has not been terminated [11].

Alcohol-use disorders accounted for about 10% of all mental illnesses, with 214.4/100,000 in 2016 in disability-adjusted life years (DALY) worldwide [12]. Although alcohol-use disorders have decreased slightly compared to the past, there are still psychiatric health problems along with depression and anxiety disorders. The problem of substance abuse is particularly problematic because it also increases the risk of developing various diseases and consequently increases the burden of disease. Alcohol abuse is known to result in a loss of about a quarter of the world’s DALY, along with smoking and drug abuse [13]. However, there are only a few recent studies on precarious work related to alcohol use, especially among younger age groups. In 2021, Shields et al. systematically collected relatively recent studies on working conditions measured using mental health evaluation tools and conducted quality evaluations. Among the studies collected, there were only four studies related to labor contracts; none of them used alcohol problems as an outcome variable [14]. Therefore, this study aims to explore how precarious employment among young age groups affects alcohol-use disorders.

## 2. Materials and Methods

### 2.1. Data Description

This study used data from the Youth Panel 2007 (YP2007), a longitudinal and annual follow-up survey conducted by the Korea Employment Information Service and funded by the Ministry of Employment and Labor. The survey consisted of a representative sample of Korean youth aged 15–29 years as the initial wave in 2007 and collected information on their school life, socioeconomic activities, and household background to develop employment policies that would help resolve youth unemployment and related problems.

For this survey, trained interviewers carried out computer-assisted personal interviews through face-to-face interviews with participants where responses were captured using laptop computers. At the baseline in 2007, a total of 10,206 participants were enrolled, and a follow-up was done annually. In 2015, the size of YP2007 was expanded by adding 3516 youths aged 15–22 years to Wave 9 to resolve the problem of lack of youth representativeness due to the increasing age of the original sample group. Since this study used Wave 10 (2016) as the baseline survey, those aged 15–29 years in the initial wave in 2007 and those aged 15–22 years added in 2015 had ages from 18 to 38 years old in 2016. The most recent survey round Wave 14 (2020) and is currently in progress. The questionnaire on alcohol consumption, including CAGE, was introduced at Wave 10. Because there was insufficient information on effect size from previous studies, we tried to include all the eligible cases in this study.

### 2.2. Participants

**Figure 1:**
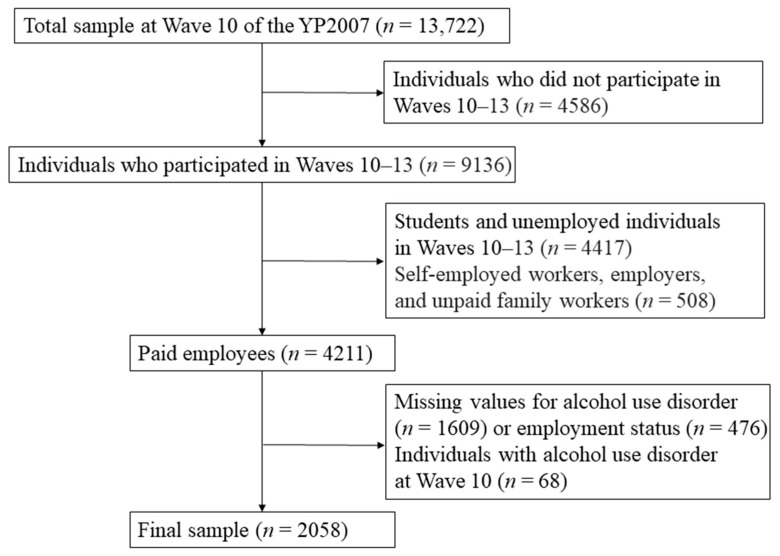
The study population using Waves 10–13 of the YP2007 survey.

Figure 1 shows a flowchart of the selection criteria used in this study. Because the information on alcohol-use disorder was collected from Wave 10, Waves 10–13 (2016–2019) were used to analyze the four-year follow-up data. Participants who did not have any information (loss of follow-up) in this period were not included. Of the available population, 9136 participants who completed Waves 10–13 were considered eligible. Because changes in employment status were the main issue of this study, students and unemployed individuals (*n* = 4417) were excluded. After that, self-employed workers, employers, and unpaid family workers (*n* = 508) were excluded because their characteristics are somewhat different from those of wage workers [15]. Among them, those with missing values for alcohol-use disorder (*n* = 1609) or employment status (*n* = 476) were also excluded. Finally, after excluding individuals with alcohol-use disorder (*n* = 68) at Wave 10, to focus on newly developed alcohol-use disorder, 2058 young employees were selected as the final sample.

### 2.3. Measurements

#### 2.3.1. Precarious Employment

During the four years between Waves 10 and 13, precarious employment (PE) was defined in a consistent manner. In this study, temporary or non-standard employment was utilized as a proxy measure for job insecurity in PE. Therefore, we defined PE based on the following three items that defined employment status to reflect the various aspects of PE: (1) “What type of employment contract do you have in your current job?” (temporary or daily vs. permanent employment); (2) “What is your employment status in your current job?” (part-time vs. full-time employment); and (3) “What type of employment do you have in your current job?” (other various types of work with characteristics of non-standard employment, including fixed-term workers, temporary workers, workers in special employment types, home-based workers, dispatched workers, subcontract workers, on-call workers, and part-time workers vs. standard workers). Individuals who did not fall into any of these categories were defined as full-time permanent workers.

The exposure variable was the duration of PE from Wave 10 to Wave 13 (four years), which was divided into three groups: (1) none (reference group in full-time permanent employment for 4 years); (2) PE for one year; and (3) PE for 2–4 years.

#### 2.3.2. CAGE (Alcohol-Use Disorder)

The CAGE (mnemonic for Cut-down, Annoyed, Guilty, and Eye-opener) is a self-report measure for alcohol-use problems. The CAGE is an acronym for four brief questions: (1) Have you ever felt you should cut down on your drinking?; (2) Have people annoyed you by criticizing your drinking?; (3) Have you ever felt bad or guilty about your drinking?; and (4) Have you ever had a drink first thing in the morning (eye-opener) to steady your nerves or to get rid of a hangover? The CAGE is considered an important measure of alcoholism even though it does not assess alcohol consumption directly [16]. Its succinctness and applicability for various clinical settings make it very attractive as a screen for alcohol-use disorders in the primary care setting [17]. We calculated the total CAGE scores, which are the total number of affirmative response(s) to the four questions, provided for each “yes” response. Alcohol-use disorders were defined using the commonly recommended cutoff value of two, indicating two or more questions were answered “yes” [18,19].

#### 2.3.3. Covariates

Information on the following variables was investigated as covariates at baseline: age (20s or younger, or 30s), sex, marital status (married or other status (single, divorced, widowed, separated)), education level (high school or below, college or above), weekly working hours (35–40 h, 40–52 h, ≥52 h), company size (less than 100 workers, 100 workers or more), binge alcohol drinking, self-rated health (good or poor), and employment status (full-time permanent or precarious). Binge drinking was defined as the intake of ≥7 drinks for men or ≥5 drinks for women on a single occasion at least twice a week [20]. Self-rated health (SRH) is a 5-point Likert scale, rated as “very good”, “good”, “fair”, “poor” and “very poor”; the latter three are classified as poor SRH [21].

### 2.4. Statistical Analysis

In this study, all analyses were stratified according to sex. Chi-square tests were performed to compare the demographic characteristics between permanent employment and PE. To reveal the relationship between PE and new cases of alcohol-use disorder at Wave 13, alcohol-use disorder at the baseline survey was excluded. Even though the participants were followed up for four years, the data collection was performed using a panel design; that is, recording of information at regular intervals, not an exact point of the event. Therefore, we calculated odds ratios (ORs) rather than hazard ratios or risk ratios. ORs and 95% confidence intervals (CIs) for newly developed alcohol-use disorders according to the duration of PE were obtained using multiple logistic regression analyses. The model was adjusted for age group, education level, and self-rated health, which were associated with alcohol dependence in previous studies, in a manner of forward selection [22,23]. All data analyses were performed using SAS version 9.4 (SAS Institute Inc., Cary, NC, USA), and *p*-values of less than 0.05 were considered to indicate statistical significance.

## 3. Results

Table 1 describes the demographic characteristics of the study participants and shows the associations between employment status and the baseline characteristics of young adults. Among the 2058 participants, the proportion of men in their 30s was relatively high (53.06% and 65.05%, respectively). Most of the participants were unmarried (69.14%) and had educational levels above college (80.56%). Young workers with PE were more likely to be younger, women, have a marital status other than “married”, have a lower education and income level, have longer working hours, and be in a smaller company. Binge drinking and SRH were not significantly associated with PE. The characteristics stratified with sex were described in Tables S1 and S2.

**Figure 2:**
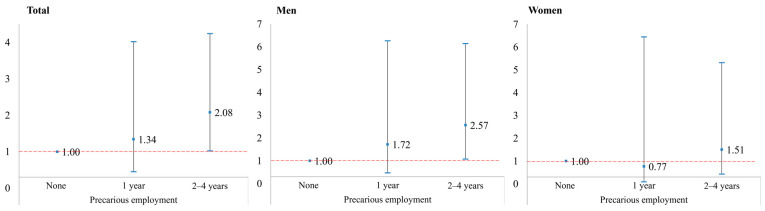
Fully adjusted odds ratios with 95% confidence intervals for CAGE as a screen for alcohol-use disorders according to precarious employment.

Table 2 depicts the relationship between the duration of PE and newly detected alcohol-use disorders at Wave 13 based on CAGE as a screening tool. In the entire study population and among male young adults, the proportion of newly developed alcohol-use disorders increased as the duration in PE increased. ORs and 95% CIs for alcohol-use disorders according to PE showed similar results after adjustment of age group, education level, binge drinking, and self-rated health. In the entire population, PE for 2–4 years (OR 2.08, 95% CI 1.02–4.24) showed a significantly increased risk of alcohol-use disorder compared with the full-time permanent sustained group, after full adjustments. Among young male adults, PE for 2–4 years (OR 2.57, 95% CI 1.07–6.14) also showed a significantly increased risk of alcohol-use disorder. Although the prevalence of alcohol-use disorders was highest in groups with PE for 2–4 years among female young adults, no significant association between alcohol-use disorders and PE was found (Figure 2).

**Table 2:** Logistic regression models for CAGE as a screen for alcohol-use disorders according to PE.

	*n* (%) *	**Crude**	**Model 1 ^a^**	**Model 2 ^b^**
**Total**	36/1506 (2.39)			
Full-time permanent sustained	18/971 (1.85)	1.00 (reference)	1.00 (reference)	1.00 (reference)
PE for 1 year	4/162 (2.47)	1.34 (0.45–4.01)	1.33 (0.44–3.98)	1.34 (0.45–4.02)
PE for 2–4 years	14/373 (3.75)	2.07 (1.02–4.20)	2.05 (1.01–4.17)	2.08 (1.02–4.24)
AIC		342.12	343.94	340.62
**Men**	24/1071 (2.24)			
Full-time permanent sustained	11/695 (1.58)	1.000 (reference)	1.000 (reference)	1.000 (reference)
PE for 1 year	3/114 (2.63)	1.68 (0.46–6.12)	1.71 (0.47–6.24)	1.72 (0.47–6.26)
PE for 2–4 years	10/262 (3.82)	2.47 (1.04–5.88)	2.55 (1.07–6.09)	2.57 (1.07–6.14)
AIC		231.71	232.52	233.16
**Women**	12/435 (2.76)			
Full-time permanent sustained	7/276 (2.54)	1.000 (reference)	1.000 (reference)	1.000 (reference)
PE for 1 year	1/48 (2.08)	0.82 (0.10–6.80)	0.75 (0.09–6.25)	0.77 (0.09–6.44)
PE for 2–4 years	4/111 (3.60)	1.44 (0.41–5.01)	1.47 (0.42–5.15)	1.51 (0.43–5.31)
AIC		115.43	112.21	112.18

PE: precarious employment. ^a^ Model 1 adjusted for age group and at baseline. ^b^ Model 2 adjusted for age group, education level, and self-rated health at baseline. * Proportion of new cases of alcohol use-disorders at Wave 13 of YP2007.

## 4. Discussion

This study confirmed that PE increases the risk of alcohol-use disorders. Because of the small proportion of alcohol-use disorder cases in the data, the ORs could be close to the amount of actual risk. It was clear that the longer the period of PE, the higher the risk. In Korea, since the 1997 financial crisis, precarious employment has increased significantly, and inequality and poverty have intensified due to structural changes in the labor market; this, in part, explains the growing alcohol consumption trend and associated problems in the society [24]. This study also highlights the deepening inequality and poverty, which was pointed out by previous studies; it shows that vulnerability to psychiatric problems, such as alcohol dependence, can be explained as a mechanism of the pathways of the social phenomena. In other words, the results of this study can be extended from the psychological symptoms of PE to how change in employment type affects alcoholism, one of the actual behavioral changes.

Several cross-sectional studies on similar topics have reported inconsistent relationship between alcohol-use disorder and employment status [25,26]. However, the results of this present study are consistent with those of the 1998 National Longitude Survey of Youth in the United States, which reported that the continuation of underemployment, such as part-time or low-waged employment, increases the risk of alcohol abuse among young people by 1.83 times compared to the continuation of adequate employment similar to full-time permanent [27]. In addition, job security, which is one of the attributes of PE, showed negative associations with alcohol or drug abuse, smoking habit, and mental health issues such as depression and anxiety [28]. On the other hand, full-time employees have better health habits, such as lower levels of alcohol consumption, smoking, and unhealthy eating habits, and higher levels of physical activity than underemployed workers [29]. The study results further solidify the hypothesis that unhealthy behavior increases due to precarious work revealed by previous studies.

It should also be pointed out that psychiatric changes caused by precarious work affected the two genders differently. The risk of alcohol-use disorders due to precarious work identified in this study was significantly higher in men than in women. In a systematic review of the areas of job stress related to major depression, the association with the occurrence of depression episodes related to high demand and low vision latency was more clearly observed in men [3]. As such, actual behavioral changes caused by occupational vulnerable factors are thought to be more prominent in men than in women. As a result of this study, studies related to stress and drinking behavior in adolescents reported that alcohol misuse due to occupational stress was more prominent in men [30]. Various reasons can explain the differences in drinking behavior between men and women: social activities, social participation roles, physiological factors, and biological vulnerabilities [31]. Further and more detailed research is needed to show why these differences occur. Macmillan and Shanahan recently presented models related to unstable labor and depressive symptoms, where they suggested that working conditions, including unstable labor, induce ‘social marginality’, which has social failure, social integration, and social capital as pathways [32]. There is also a possibility that there will be gender differences in the role of these pathways. However, in this study, there is a limitation in that it was not possible to verify these routes. Phenomenally, unhealthy behavior is prominent in men, so it can be a reference for determining intervention priorities.

Interventions through occupational stress relief considering job demand and control are not a sustainable solution. Nielsen et al. tried to improve workers’ alcohol consumption habits through intervention in job control and demand, which are areas of job stress, but in a two-year follow-up, such attempts were found to have little effect [33]. Therefore, supporting programs that will improve job security should be considered as a priority intervention, which would be more difficult to achieve in effect than general occupational stress-relief programs.

This study confirmed a temporal relationship between the duration of precarious work and the risk of alcohol disorder; the longer the period of precarious work, the higher the risk of alcohol-use disorders. This is a research design that can overcome the inconsistency that appeared in previous cross-sectional studies. However, even though it was tracking data over time, there was a limitation, as the time points were fixed because it was a panel survey that only tracks once a year. Moreover, even though we considered age in the regression models, the age of first employment should have been considered because earlier participation in the labor market may be associated with alcohol-use habits. The YP2007 does not have information on the first employment in life; however, we could consider the age of the first year at the current job. In a model adjusting it, we could not observe statistical significance. The age at the first employment might have a mediating effect between alcohol-use disorder and precarious work. We might expect the mediating effect from canceling the significance; however, we could not exclude exogenous error. Extended and more observations considering the mediating effect is needed in future analyses. Additionally, we confirmed a limited number of alcohol-use disorder cases (2.39%), which led to wide confidence intervals. Even though screening alcohol-use disorder was defined by CAGE ≥ 2, we tried a sensitivity analysis by using an operative definition of CAGE ≥ 1 (Table S3). As excluding participants of CAGE ≥ 1 initially, 118 out of 1360 (8.68%) were new CAGE ≥ 1 cases in the follow-up period. We confirmed statistically high odds ratios with PE, even though there was no dose–response relationship. We still did not observe significant results among women. As mentioned earlier, there was a limitation in the data, in that these were not sufficiently investigated to identify the mechanistic pathways towards alcohol-use disorder. In addition, use of CAGE was also a limitation as the exact diagnosis was not used as a dependent variable.

## 5. Conclusions

In conclusion, this study suggested that the longer the unstable labor, the higher the risk of alcohol-dependence disorder, and showed that the tendency was stronger in men. Further studies should be conducted on the factors showing this tendency in other groups, such as other than young ages or other societies. In addition, because people engaged in PE are vulnerable to alcohol-use disorders, policy programs focusing on them are needed.

## Figures and Tables

**Table 1:** Characteristics of all the study participants at the baseline (Wave 10) and differences between employment status.

**Characteristics**	**Total [*n* (%)]**	**Employment Status [*n* (%)]**		***p*-Value** *
		**Permanent**	**Precarious**	
	2058 (100)	1579 (76.72)	479 (23.28)	
**Age**				
18–29	966 (46.94)	709 (73.40)	257 (26.60)	0.001
30–38	1092 (53.06)	870 (79.67)	222 (20.33)	
**Sex**				
Men	1339 (65.06)	1051 (78.49)	288 (21.51)	0.010
Women	719 (34.94)	528 (73.44)	191 (26.56)	
**Marital status**				
Married	635 (30.86)	523 (82.36)	112 (17.64)	<0.0001
Other	1423 (69.14)	1056 (74.21)	367 (25.79)	
**Education level**				
High school and below	400 (19.44)	280 (70.00)	120 (30.00)	<0.0001
College and above	1658 (80.56)	1299 (78.35)	359 (21.65)	
**Weekly working hours**				
35–40 h	1152 (57.09)	922 (80.03)	230 (19.97)	0.009
40–52 h	700 (34.69)	534 (76.29)	166 (23.71)	
≥52 h	166 (8.23)	117 (70.48)	49 (29.52)	
**Income level**				
<200,000,000 KR Won	514 (25.43)	313 (60.89)	201 (39.11)	<0.0001
≥200,000,000 KR Won	1507 (74.57)	1242 (82.42)	265 (17.58)	
**Company size**				
<100 workers	1313 (63.80)	972 (74.03)	341 (25.97)	<0.0001
≥100 workers	745 (36.20)	607 (81.48)	138 (18.52)	
**Binge drinking** ^†^				
No	1829 (88.87)	1401 (76.60)	428 (23.40)	0.703
Yes	229 (11.13)	178 (77.73)	51 (22.27)	
**Self-rated health**				
Good	1817 (88.29)	1405 (77.33)	412 (22.67)	0.077
Poor	241 (11.71)	174 (72.20)	67 (27.80)	

* *p*-values from chi-square tests; ^†^ Binge drinking was defined as the intake of ≥7 drinks for men or ≥5 drinks for women on a single occasion at least twice a week.

## Data Availability

The english version of YP2007 is available in the website: https://survey.keis.or.kr/eng/index.jsp.

## Supplementary Materials

The following supporting information can be downloaded at: https://www.mdpi.com/article/10.3390/ijerph19127380/s1, Table S1: Characteristics of male participants at the baseline (Wave 10) and differences between employment status; Table S2: Characteristics of female participants at the baseline (Wave 10) and differences between employment status; Table S3: Logistic regression models for CAGE ≥ 1 as a screen for alcohol use disorders according to PE.
