# Supplementary material for: Alcohol Abuse Associated with Accumulated Periods of Precarious Employment: A Four-Year Follow-Up Study of a Young Population in Korea
Source: Int J Environ Res Public Health. 2022 Jun 16;19(12):7380. doi: 10.3390/ijerph19127380 (PMC9223578; doi:10.3390/ijerph19127380)
Supplement: Supplementary file 1 [file ijerph-19-07380-s001.zip › ijerph-1724544-supplementary.pdf]

**Supplementary Table S1.** Characteristics of male participants at the baseline (Wave 10) and differences between employment status.

| Characteristics | Total [N (%)] | Employment status [N (%)] | | p-value* |
|---|---|---|---|---|
| | | Permanent | Precarious | |
| | 1339 (100) | 1051 (78.49) | 288 (21.51) | |
| **Age** | | | | |
| 18–29 | 939 (70.13) | 762 (72.5) | 177 (61.46) | <.001 |
| 30–38 | 400 (29.87) | 289 (27.5) | 111 (38.54) | |
| **Marital status** | | | | |
| Married | 503 (37.57) | 425 (40.44) | 78 (27.08) | <.001 |
| Other | 836 (62.43) | 626 (59.56) | 210 (72.92) | |
| **Education level** | | | | |
| High school and below | 299 (22.33) | 214 (20.36) | 85 (29.51) | 0.001 |
| College and above | 1040 (77.67) | 837 (79.64) | 203 (70.49) | |
| **Weekly working hours** | | | | |
| 35–40h | 715 (54) | 588 (56.05) | 127 (46.18) | 0.008 |
| 40–52h | 475 (35.88) | 364 (34.7) | 111 (40.36) | |
| ≥52h | 134 (10.12) | 97 (9.25) | 37 (13.45) | |
| **Income level** | | | | |
| <200,000,000 KR Won | 213 (16.14) | 117 (11.27) | 96 (34.04) | <.0001 |
| ≥200,000,000 KR Won | 1107 (83.86) | 921 (88.73) | 186 (65.96) | |
| **Company size** | | | | |
| <100 workers | 833 (62.21) | 627 (59.66) | 206 (71.53) | <.001 |
| ≥100 workers | 506 (37.79) | 424 (40.34) | 82 (28.47) | |
| **Binge drinking†** | | | | |
| No | 1156 (86.33) | 908 (86.39) | 248 (86.11) | 0.902 |
| Yes | 183 (13.67) | 143 (13.61) | 40 (13.89) | |
| **Self-rated health** | | | | |
| Good | 1197 (89.4) | 945 (89.91) | 252 (87.5) | 0.238 |
| Poor | 142 (10.6) | 106 (10.09) | 36 (12.5) | |

*P values from chi-square tests

†Binge drinking was defined as the intake of ≥7 drinks for men or ≥5 drinks for women on a single occasion at least twice a week.

**Supplementary Table S2.** Characteristics of female participants at the baseline (Wave 10) and differences between employment status.

| Characteristics | Total [N (%)] | Employment status [N (%)] | | p-value* |
|---|---|---|---|---|
| | | Permanent | Precarious | |
| | 719 (100) | 528 (73.44) | 191 (26.56) | |
| **Age** | | | | |
| 18–29 | 408 (56.75) | 311 (58.9) | 97 (50.79) | 0.052 |
| 30–38 | 311 (43.25) | 217 (41.1) | 94 (49.21) | |
| **Marital status** | | | | |
| Married | 132 (18.36) | 98 (18.56) | 34 (17.8) | 0.816 |
| Other | 587 (81.64) | 430 (81.44) | 157 (82.2) | |
| **Education level** | | | | |
| High school and below | 101 (14.05) | 66 (12.5) | 35 (18.32) | 0.047 |
| College and above | 618 (85.95) | 462 (87.5) | 156 (81.68) | |
| **Weekly working hours** | | | | |
| 35–40h | 437 (62.97) | 334 (63.74) | 103 (60.59) | 0.209 |
| 40–52h | 225 (32.42) | 170 (32.44) | 55 (32.35) | |
| ≥52h | 32 (4.61) | 20 (3.82) | 12 (7.06) | |
| **Income level** | | | | |
| <200,000,000 KR Won | 301 (42.94) | 196 (37.91) | 105 (57.07) | <.0001 |

| | | | | |
|---|---|---|---|---|
| ≥200,000,000 KR Won | 400 (57.06) | 321 (62.09) | 79 (42.93) | |
| **Company size** | | | | |
| <100 workers | 480 (66.76) | 345 (65.34) | 135 (70.68) | 0.180 |
| ≥100 workers | 239 (33.24) | 183 (34.66) | 56 (29.32) | |
| **Binge drinking**[†] | | | | |
| No | 673 (93.60) | 493 (93.37) | 121 (94.24) | 0.674 |
| Yes | 46 (6.40) | 35 (6.63) | 11 (5.76) | |
| **Self-rated health** | | | | |
| Good | 620 (86.23) | 460 (87.12) | 160 (83.77) | 0.249 |
| Poor | 99 (13.77) | 68 (12.88) | 31 (16.23) | |

[*]P values from chi-square tests

[†]Binge drinking was defined as the intake of ≥7 drinks for men or ≥5 drinks for women on a single occasion at least twice a week.

**Supplementary Table S3.** Logistic regression models for CAGE ≥1 as a screen for alcohol use disorders according to PE.

| | N (%)[*] | Crude | Model 1[b] | Model 2[b] |
|---|---|---|---|---|
| **Total** | 118/1360 (8.68) | | | |
| Full-time permanent sustained | 59/873 (6.76) | 1.00 (reference) | 1.00 (reference) | 1.00 (reference) |
| PE for 1 year | 20/154 (12.99) | 2.06 (1.20–3.53) | 2.07 (1.21–3.54) | 2.13 (1.24–3.69) |
| PE for 2–4 years | 39/333 (11.71) | 1.83 (1.20–2.80) | 1.84 (1.20–2.82) | 1.85 (1.20–2.86) |
| **Men** | 87/955 (9.11) | | | |
| Full-time permanent sustained | 44/619 (7.11) | 1.00 (reference) | 1.00 (reference) | 1.00 (reference) |
| PE for 1 year | 15/110 (13.64) | 2.08 (1.11–3.89) | 2.11 (1.13–3.94) | 2.29 (1.21–4.32) |
| PE for 2–4 years | 28/226 (12.39) | 1.95 (1.18–3.23) | 1.92 (1.16–3.17) | 1.95 (1.16–3.27) |
| **Women** | 31/405 (7.65) | | | |
| Full-time permanent sustained | 15/254 (5.91) | 1.00 (reference) | 1.00 (reference) | 1.00 (reference) |
| PE for 1 year | 5/44 (11.36) | 2.04 (0.70–5.94) | 1.92 (0.65–5.64) | 1.78 (0.60–5.30) |
| PE for 2–4 years | 11/107 (10.28) | 1.83 (0.81–4.12) | 1.87 (0.82–4.25) | 1.79 (0.78–4.08) |

PE: precarious employment. [a] Model 1 adjusted for age group and at baseline. [b]Model 2 adjusted for age group, education level, and self-rated health at baseline.

* Proportion of new cases of alcohol use disorders defined based on CAGE ≥1 at Wave 13 of YP2007.
